# An assessment of the content of discharge summaries at Mount Carmel Hospital, Malta

**DOI:** 10.1192/j.eurpsy.2021.2087

**Published:** 2021-08-13

**Authors:** E. Camilleri, G. Grech, Y. Farrugia, J. Padovani

**Affiliations:** Psychiatry, Mount Carmel Hospital, Attard, Malta

**Keywords:** discharge summaries, quality improvement, communication

## Abstract

**Introduction:**

Discharge summaries are the mainstay of intra and inter-departmental communication, ensuring continuity of care. Local instructions fail to provide clear guidance to foundation doctors to ensure standardised discharge summaries.

**Objectives:**

The audit aimed to assess the inclusion of information within discharge summaries at Mount Carmel Hospital, Malta. A secondary objective was to update the current online discharge summary framework.

**Methods:**

Stratified random sampling was used to select 120 discharge summaries, issued between October 2018 and September 2019. These were chosen out of a total of 956 discharge summaries issued during the period. The inclusion of information was analysed against the National Standard for Patient Discharge Summary Information issued by the Health Information and Quality Authority, Ireland. Data was collected and grouped into seven categories each containing multiple data points.

**Results:**

Patient details were present in all discharge summaries while no details relating to the primary care healthcare professional were documented. The average information inclusion rate regarding admission, discharge and medications was 85%. Average clinical information was documented in 50% while that of future management and person completing discharge summary was found in 41% and 28% respectively (as per Table 1). Encouragingly, discharge summaries contained mandatory information more frequently than conditional or optional information.

**Figure:**
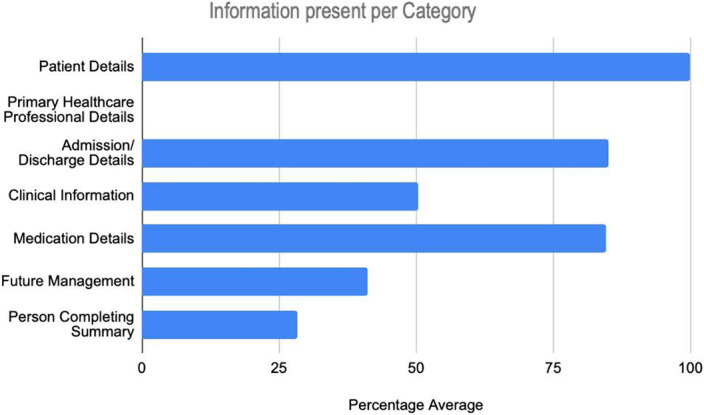

**Conclusions:**

This audit has identified deficiencies in current discharge summary practices and gives recommendations for the development of local guidelines.

**Disclosure:**

No significant relationships.

